# The priority of liver resection compared with transarterial chemoembolization in hepatocellular carcinoma at BCLC B1 stage: A single-center experience

**DOI:** 10.3389/fsurg.2022.920976

**Published:** 2022-11-09

**Authors:** Peng-Sheng Yi, Jun-Ning Liu, Yong Li, Bin Wu, Jian-Shui Li

**Affiliations:** Department of Hepato-Biliary-Pancreas II, Affiliated Hospital of North Sichuan Medical College, Nanchong, China

**Keywords:** hepatocellular carcinoma, BCLC B1 stage, liver resection, transarterial chemoembolization, safety

## Abstract

**Background:**

This study aimed to compare the efficacy of liver resection (LR) and transarterial chemoembolization (TACE) in the treatment of Barcelona Clinic Liver Cancer B1 (BCLC B1) hepatocellular carcinoma.

**Methods:**

A total of 65 patients with BCLC B1 were divided into the radical (LR group) and TACE groups. Survival analysis was performed using the Kaplan–Meier method. Univariate and multivariate analyses were carried out, and the prognostic factors for survival outcomes were identified using Cox proportional analysis.

**Results:**

The 1-, 3-, and 5-year survival rates and the 1-, 3-, and 5-year progression-free survival (PFS) rates in the LR group (*P* = 0.036) were significantly higher than those in the TACE group (*P* = 0.027). Results of the multivariate analysis demonstrated that tumor distribution (both lobes vs. semi-liver) and treatment strategy (LR vs. TACE) were independent risk factors for the overall survival (OS) [hazard ratios (HRs): 3.926 and 0.479; *P* < 0.05] and PFS (HR: 3.336 and 0.465, *P* < 0.05). LR was associated with increased OS and PFS compared with TACE in patients with BCLC B1 hepatocellular carcinoma.

## Introduction

Liver cancer is the third leading cause of cancer-related deaths worldwide, and the second most lethal cancer in China (1, 2). Hepatocellular carcinoma (HCC) accounts for 75%–85% of primary liver cancer cases (2). With the advancement of treatment and surveillance strategies, the survival rate of patients with HCC has increased in the past few decades, but remains unsatisfactory. Early detection and development of novel treatment strategies are critical to improving the patients’ prognosis. The Barcelona Clinic Liver Cancer (BCLC) staging system is commonly used for determining the treatment strategy of patients with HCC. According to this staging system, radical treatment strategies should be applied to very early and early stage HCC (3). However, most patients are already diagnosed at the intermediate or advanced stages of the disease at the initial visit.

Patients with BCLC B stage HCC presented with large differences in tumor burden, liver function and general conditions. Based on the BCLC recommendations, transarterial chemoembolization (TACE) is the first-line treatment for BCLC B stage HCC. However, not all patients with intermediate-stage HCC benefit from TACE. In addition, the BCLC staging system requires further modification. Bolondi et al. proposed a substage system for BCLC B stage in 2012 (4), which divides intermediate-stage HCC into four substages and provides first-line and alternative treatment strategies for different groups. In recent years, subsequent studies have been performed to validate this system (5–7). According to the Bolondi system, liver resection (LR) is no longer recommended as the primary option. However, its efficacy for patients with intermediate-stage HCC has been validated by several studies (8–11). Thus, the indications of LR for BCLC B stage HCC need further expansion (12–14). Kudo et al. proposed a modified subclassification system similar to the Bolondi criteria (Table 1) in 2015 (Kinki criteria) (7). The difference was that they simplified the clinical parameters and provided different treatment strategies for patients with BCLC B1. According to the modified criteria, patients with the B1 stage should undergo radical treatment, such as resection or ablation, and TACE is recommended as a secondary option.

**Table 1:** Subclassification of BCLC B stage hepatocellular carcinoma.

Subclassification	Bolondi criteria				Kinki criteria			
	B1	B2	B3	B4	B1	B2	B3a	B3b
Child–Pugh score	5–7	5–6	7	8–9	5–7	5–7	8–9	
Beyond Milan and within up-to-7	In	Out	Out	Any	In	Out	Any	
							In	Out
ECOG PS	0	0	0	0–1^a^				
PVT	No	No	No	No				

BCLC, Barcelona clinic liver cancer; ECOG PS, Eastern Cooperative Oncology Group Performance Score; PVT, portal vein thrombosis.

^a^ The 2022 updated BCLC strategy patients with PS 1 as advantage (15).

BCLC B1 stage is characterized by compensated cirrhosis and preserved liver function, a Child–Pugh score of 5–7, a tumor burden beyond the Milan criteria, a tumor burden within the up-to-7 criteria (the sum of the largest tumor size and tumor number is not less than 7), and a completely preserved Eastern Cooperative Oncology Group Performance Score (ECOG PS). The Bolondi system recommends TACE as the primary option and liver transplantation or TACE + ablation as an alternative for those with the B1 stage. Ciria et al. performed a retrospective study in 80 patients with BCLC B stage disease (16), and reported the 5-year survival rates were not significantly different between patients who underwent LR and those who underwent TACE. However, the survival rate of patients with B1 stage who underwent LR was significantly better than those with B2, B3, and B4 stages. Thus, the treatment strategy for B1 stage disease remains controversial. The present study aimed to compare the efficacy of LR and TACE in patients with B1 stage. Results were reported in accordance with the Strengthening the Reporting of Observational Studies in Epidemiology reporting checklist.

## Patients

**Figure 1:**
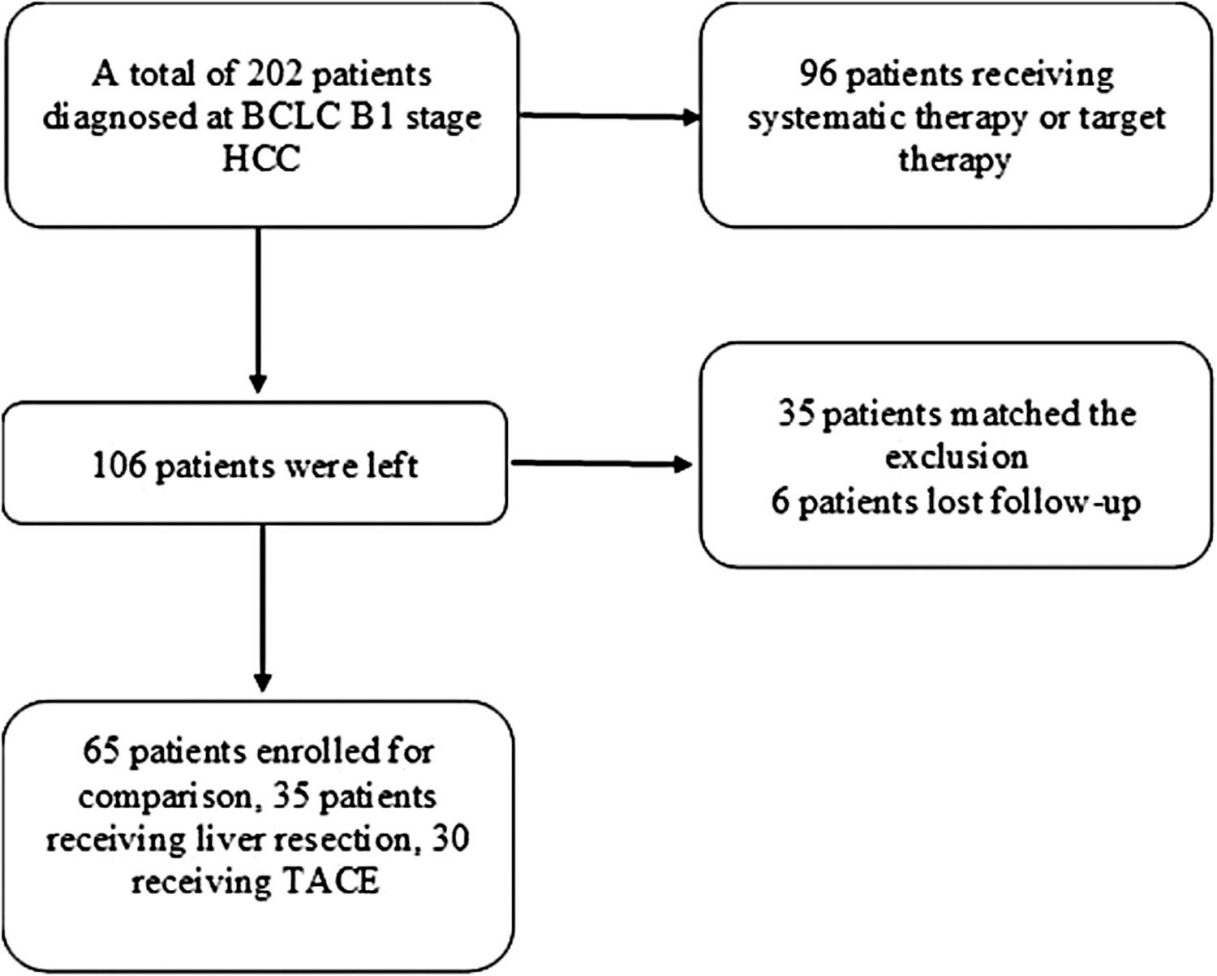
Flow chart of patient selection.

A total of 65 patients diagnosed with BCLC B1 at the Affiliated Hospital of North Sichuan Medical College between February 2010 and October 2015 were enrolled in the present study. BCLC B1 stage HCC was defined based on the features of patients such as the occurrence of compensated cirrhosis and preserved liver function, a Child–Pugh score of 5–7, occurrence of tumors within the up-to-7 criteria, and a completely preserved ECOG PS. According to different therapeutic strategies, the retrospectively recruited patients were classified into two groups: the radical LR group and the TACE group. The treatment strategies for patients were selected based on the decisions of multidisciplinary teams. The indications for LR included resectable tumors, appropriate residual liver volume, Child–Pugh score of 5–7 without ascites and hypersplenism. The criteria for TACE included a Child–Pugh score of 5–7 and the absence of massive ascites. Moreover, patients who refused to undergo LR were treated with TACE. In order to make a definite clinical diagnosis, all patients underwent radiological examinations such as computed tomography (CT) or magnetic resonance imaging (MRI), and biopsy was performed when the diagnosis was not certain. Patients with (i) a diagnosis of BCLC B1 stage HCC; (ii) good liver function (Child–Pugh score of 5–7); and (iii) good performance status (PS 0) were included in the study. Meanwhile, patients (i) aged <18 years or ≥75 years old; (ii) who received any previous systemic therapy (chemotherapy or target therapy); (iii) previously or currently diagnosed with other malignant tumors; (iv) with active cardiopulmonary disease or infection, except for hepatitis B virus (HBV); and (v) with incomplete data or who were lost to follow-up were excluded. This study was approved by the Ethics Committee of Affiliated Hospital of North Sichuan Medical College, and written informed consent was obtained from all patients prior to the beginning of the study. A flow chart showing the patient selection process is shown in Figure 1.

## Surgical procedures

The surgical procedures involved both laparoscopic and open radical LR, including nonanatomic regional resection, segment resection, and lobe resection. The resection line and tumor lesions were identified using intraoperative ultrasound, and patients with positive resection margins were excluded from the present study. Hemihepatectomy was performed as a routine anatomic regional resection with selective hepatic arteries and portal venous amputation. Irregular hepatectomy especially in the right hemi-liver and middle lobe was performed as nonanatomic regional resection, and the Pringle maneuver was performed for 15–20 min with a 5 min clamp-free interval to block the blood flow to the liver. LR was performed using a Cavitron Ultrasonic Surgical Aspirator or harmonic scalpel, while coagulation was performed using a bipolar coagulator. All resected specimens were submitted for histological examination.

All procedures were performed with curative intent, with the aim of R0 resection. The tumor characteristics were determined, and a positive margin was defined as a tumor-free margin of <1 mm. All surgical procedures were performed by surgeons with extensive experience, and the resection line and tumor lesions were identified *via* intraoperative ultrasound. However, positive margins are an inherent problem in LR. Preoperative assessment and precision operation are sufficient to achieve negative resection margins. If a positive resection margin occurs, the procedures performed on the patients are not meaningful, and the long-term survival benefit for patients with resectable HCC is not possible. Since the application of three-dimensional CT reconstruction and intraoperative ultrasound, the relationship between tumor lesions and intrahepatic ducts can be recognized, and seldom positive resection margins have occurred in our medical center in recent years.

Considering that this study aimed to compare the survival benefit of radical LR with that of TACE for BCLC B1 HCC, solitary cases with positive resection margins were excluded.

### TACE

Preoperative assessment was performed prior to TACE to determine the liver function, renal function, blood cell count, and PS. A 5-fluorouracil infusion catheter was selectively inserted into the tumor-feeding hepatic arteries. An emulsion of epirubicin (20–40 mg; Pharmorubicin; Pfizer) and Lipiodol (2–10 ml; Guerbet) was injected into the nutrient artery and small gelatin sponge particles were used for embolization. A CT scan was performed 4 weeks after TACE to determine the effect based on the status of iodine oil deposition.

### Follow-up

All patients were contacted *via* telephone and e-mail, and the first follow-up was conducted 4 weeks after the operation. If recurrence was not detected, follow-up was performed at an interval of 2 months in the first year. If early recurrence (recurrence within 2 years after surgery) did not occur, follow-up was performed every 6 months. CT or MRI was performed to detect any recurrence or metastasis. The serum alpha-fetoprotein (AFP) level, prothrombin time, and liver function were assessed, and the HBV deoxyribonucleic acid loading was measured if an underlying HBV infection was present.

Overall survival (OS) was defined as the period from the date of treatment to the time of death or last follow-up visit, whereas progression-free survival (PFS) was defined as the period from the date of treatment to the time of disease progression. The Clavien–Dindo grading system of complications was used to assess the postoperative complications after LR or TACE for HCC patients. Follow-up was censored in December 2020.

### Statistical analysis

Categorical variables were compared using χ^2^ or Fisher's exact tests. Survival analysis was performed using the Kaplan–Meier method and the log-rank test. Univariate and multivariate Cox regression analyses were performed to identify the factors affecting the survival outcomes. Only variables with significance (*P* < 0.05) in the univariable model were included in the multivariable analysis. For all statistical analyses, a *P* value of <0.05 was considered significant. Statistical analysis was performed using the SPSS 21 software (IBM Corp.) and R-studio (version 4.2.1).

## Patient characteristics

Patient characteristics are presented in Table 2. Sixty-five patients diagnosed with BCLC B1 stage HCC between February 2010 and October 2015 were included in the present study. Among them, 57 patients were men and 8 were women, 25 were aged >60 years, 57 had HBV infection, 45 had liver cirrhosis, 18 had more than three tumors, and 8 had more than four tumors. Patients with HCC and HBV infection were treated with entecavir (0.5 mg) daily throughout their lifetime. Five patients presented with tumor lesions in both lobes, two of whom underwent right semi-hepatectomy plus regional left liver lobe resection (one tumor lesion located in the inferior left lateral lobe). The other three patients underwent anatomic segment resection plus radiofrequency ablation. Of them, two patients presented with tumor lesions distal to the liver capsule, and one patient presented with a tumor lesion proximal to the large hepatic vein. No perioperative surgery-related deaths were observed, and bile leakage, pulmonary infection, or liver failure occurred in several patients. The demographic characteristics of these two groups were comparable. Among all patients, only two developed severe postoperative complications (grade 3/4). In the TACE group, fever, nausea, and abdominal pain (which were postembolization syndrome features) were the most common complications. According to the Clavien–Dindo grading system, no significant differences in any of the complications according to grade (Table 3).

**Table 2:** Comparison of baseline characteristics of enrolled patients.

Clinicopathological factors	LR group (*n* = 35)	TACE group (*n* = 30)	*P* value
Gender			
Male	30	27	0.716
Female	5	3	
Age			
60	12	13	0.455
≤60	23	17	
HBsAg			
Positive	30	27	0.716
Negative	5	3	
AFP (ng/ml)			
>400	22	8	**0.004**
≤400	13	22	
Child–Pugh score			
7	6	4	0.937
5–6	29	26	
Liver cirrhosis			
Yes	24	21	0.901
No	11	9	
Tumor number			
≥3	10	8	0.864
=2	25	22	
Tumor number			
>3	4	4	1.000
≤3	31	26	
Largest tumor size			
>4 cm	15	12	0.816
≤4 cm	20	18	
Largest tumor size			
>3 cm	30	25	1.000
≤3 cm	5	5	
Tumor distribution			
Both lobes	5	3	0.716
Semi-liver	30	27	
Tumor capsule			
Complete	24	16	0.084
Infiltration	11	14	

HBsAg, hepatitis B surface antigen; AFP, α- fetoprotein; LR, liver resection; TACE, transarterial chemoembolization.

**Table 3:** Postoperative complications in the two patient group *n* (%).

	Total (*n* = 65)	LR group (*n* = 35)	TACE group (*n* = 30)	*P* value
Complications	13 (20.0)	8 (22.9)	5 (16.6)	0.534
Grade 1	6 (9.2)	4 (11.4)	2 (6.7)	
Grade 2	5 (7.7)	3 (8.6)	2 (6.7)	
Grade 3	1 (1.5)	0 (0)	1 (3.3)	
Grade 4	1 (1.5)	1 (2.9)	0 (0)	
Grade 5	0 (0)	0 (0)	0 (0)	0.856
Minor complications (1–2)	11 (16.9)	7 (20.0)	4 (13.3)	0.475
Major complications (3–4)	2 (3.1)	1 (2.9)	1 (3.3)	1.000

LR, liver resection; TACE, transarterial chemoembolization.

### OS analysis

**Figure 2:**
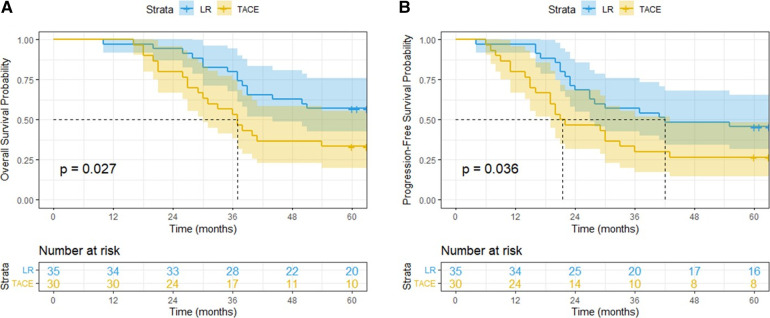
Overall survival (**A**) and progression-free survival (**B**) curves of patients with BCLC-B1 stage HCC treated by liver resection or transarterial chemoembolization.

The median follow-up time was 63 months, while the median OS time of all patients was 50 months. The median OS time in LR group was not reached, while that in TACE group, was 37 months [95% confidence interval (CI): 30.31–43.70 months; hazard ratio (HR): 0.482; 95% CI: 0.245–0.951; *P* = 0.027]. The 1-, 3-, and 5-year OS rates were 98.5%, 67.7%, and 46.2%, respectively. The 1-, 3-, and 5-year OS rates in LR group were 97.1%, 80.0%, and 57.1%, respectively. The 1-, 3-, and 5-year OS rates in TACE group were 100.0%, 53.3%, and 33.3%, respectively. Notably, patients in the LR group had better survival outcomes compared with those in the TACE group (*P* = 0.027) (Figure 2A).

**Figure 3:**
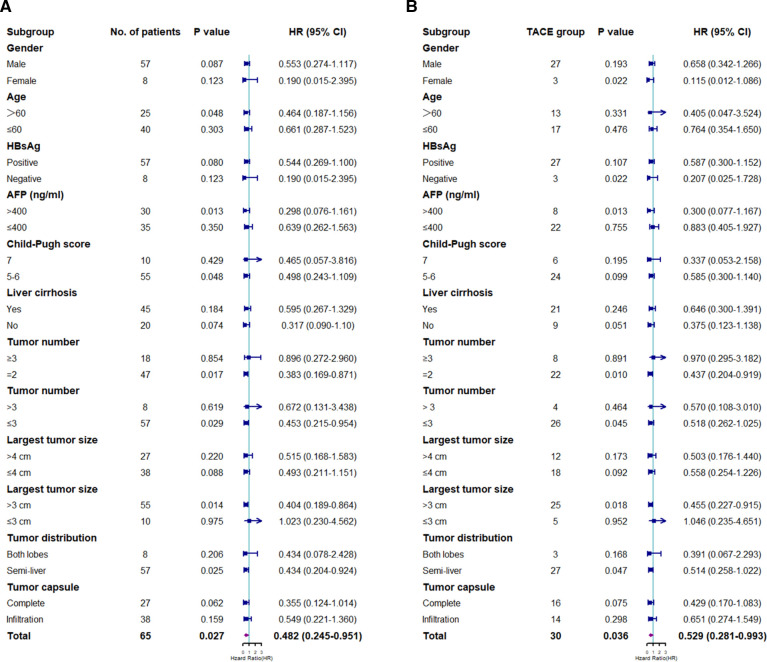
Subgroup analyses of overall survival (**A**) and progression-free survival (**B**) for prognostic factors. HR, hazard ratio; CI, confidence interval; HBsAg, hepatitis B surface antigen; AFP, *α*-fetoprotein; LR, liver resection; TACE, transarterial chemoembolization.

Subgroup analysis was performed according to the baseline characteristics. The median OS time in the LR group was significantly longer than that in the TACE group in patients aged >60 years, with a serum AFP level of >400 (ng/ml), with a Child–Pugh score of 5–6, with 2–3 tumor lesions, whose largest tumor size was >3 cm, and whose tumor lesions were located within the semi-liver (*P* < 0.05) (Figure 3A).

### PFS analysis

The median PFS time of all patients was 30 months (95% CI: 19.85–40.15 months); the median PFS time in the LR and TACE groups was not reached, while that in the TACE groups was 21 months (95% CI: 8.92–33.08 months; HR: 0.529; 95% CI: 0.281–0.993; *P* = 0.036). The 1-, 3-, and 5-year PFS rates were 89.2%, 44.6% and 36.9%, respectively. The 1-, 3-, and 5-year PFS rates in LR group were 97.1%, 57.1% and 45.7%, respectively. The 1-, 3-, and 5-year PFS rates in TACE group were 80.0%, 30.0%, and 26.7%, respectively. Notably, patients in the LR group exhibited significantly lower recurrence rates than those in the TACE group (*P* = 0.036) (Figure 2B).

Subgroup analysis was performed based on the clinicopathological characteristics, and the PFS rate in the LR group was significantly lower than that in the TACE group in female patients, patients with negative results on HBsAg test, patients with a serum AFP level of >400 (ng/ml), patients with 2–3 tumor lesions, patients whose largest tumor size was >3 cm, and patients whose tumor lesions were located within the semi-liver (*P* < 0.05) (Figure 3B).

### Univariate and multivariate analyses of risk factors for OS and PFS

A total of 12 parameters were assessed in the univariate analysis, and tumor distribution and treatment strategy were independent risk factors for OS and PFS (*P* < 0.05). The multivariate analysis demonstrated that tumor distribution (both lobes vs. semi-liver) (HR: 3.926, 95% CI: 1.659–9.292, *P* = 0.002) and treatment strategy (LR vs. TACE) (HR: 0.410, 95% CI: 0.206–0.820, *P* = 0.012) were independent risk factors for OS. Similarly, the multivariable Cox proportional hazards model for PFS identified the tumor distribution (both lobes vs. semi-liver) (HR: 3.336, 95% CI: 1.429–7.788, *P* = 0.005) and treatment strategy (LR vs. TACE) (HR: 0.465, 95% CI: 0.247–0.876, *P* = 0.018) as independent risk factors (Table 4).

**Table 4:** Univariate and multivariate analysis of OS and PFS.

Variable	OS				PFS			
	Univariate analysis		Multivariate analysis		Univariate analysis		Multivariate analysis	
	HR (95% CI)	*P* Value	HR (95% CI)	*P* Value	HR (95% CI)	*P* Value	HR (95% CI)	*P* Value
Gender								
Male	1.641 (0.502-5.342)	0.412			1.269 (0.452-3.564)	0.651		
Female								
Age								
>60	0.632 (0.309-1.291)	0.208			0.627 (0.324-1.212)	0.165		
≤60								
HBsAg								
Positive	1.751 (0.536-5.722)	0.354			0.775 (0.326-1.844)	0.564		
Negative								
AFP (ng/ml)								
>400	0.856 (0.438-1.674)	0.650			0.595 (0.315-1.125)	0.110		
≤400								
Liver cirrhosis								
Yes	1.033 (0.496-2.152)	0.932			0.820 (0.430-1.564)	0.547		
No								
Tumor number								
≥3	1.131 (0.554-2.312)	0.735			0.954 (0.478-1.905)	0.895		
=2								
Tumor number								
>3	1.743 (0.722-4.206)	0.216			1.608 (0.674-3.840)	0.285		
≤3								
Largest tumor size								
>4 cm	0.757 (0.381-1.504)	0.427			0.721 (0.381-1.362)	0.313		
≤4 cm								
Largest tumor size								
>3 cm	0.674 (0.294-1.546)	0.352			0.697 (0.308-1.577)	0.387		
≤3 cm								
Tumor distribution								
Both lobes	3.135 (1.362-7.214)	0.007	3.926 (1.659-9.292)	0.002	2.692 (1.185-6.115)	0.018	3.336 (1.429-7.788)	0.005
Semi-liver								
Tumor capsule								
Complete	0.989 (0.503-1.946)	0.975			1.239 (0.668-2.297)	0.497		
Infiltration								
Treatment Group								
LR	0.480 (0.245-0.939)	0.032	0.410 (0.206-0.820)	0.012	0.534 (0.288-0.988)	0.046	0.465 (b>0.247-0.876)	0.018
TACE								

OS, over survival; PFS, progression-free survival; HR, hazard ratio; CI; confidence interval; HBsAg, hepatitis B surface antigen; AFP, α- fetoprotein; LR, liver resection; TACE, transarterial chemoembolization.

## Discussion

According to the BCLC guidelines, intermediate-stage HCC is characterized by varied tumor burden and liver function, and TACE is recommended as the first-line treatment for this condition. However, recent studies have reported the benefits of radical treatment in patients with BCLC B stage HCC. In addition, several researchers have proposed expanding the indications for LR in the treatment of intermediate-stage HCC. Bolondi et al. introduced a substaging system for BCLC B stage in 2012 (4), which classified BCLC B into four stages. This staging system was validated in subsequent studies. Kudo et al. developed this system and proposed a new substaging system (Kinki criteria) (7). Despite the similarities with Bolondi's system, the Kinki criteria are simpler and easier to apply, and resection and ablation are included as treatment options for patients with substage B1 disease. Although BCLC B stage HCC patients had a PS of 0–1 based on previous BCLC strategy, the Bolondi's criteria stressed that the ECOG PS score for the BCLC B1 substage was 0. Therefore, only BCLC B1 stage patients with PS 0 were included and fulfilled the 2022 updated staging criteria (15). Both systems define patients with tumors within up-to-7 criteria and with good liver function as having a BCLC B1 substage; however, the treatment recommendations differ. Therefore, the present study compared the survival benefits of LR and TACE in patients with the BCLC B1 stage.

The results of the present study demonstrated that LR significantly prolonged the OS and PFS of patients with BCLC B1 stage compared with those in the TACE group. Wang et al. and Scaffaro et al. performed two studies to validate Bolondi's criteria (17, 18). These findings demonstrated that patients with BCLC B1 stage who underwent TACE and LR had median OS times of 2.4 and 2.8 years, respectively. Arizumi et al. performed a retrospective study in 2015 to validate the Kinki criteria (6). All patients who underwent TACE and were diagnosed with BCLC B1 subclass had a median OS time of 3.0 years. This group performed another study in 2016 to validate the Kinki criteria (19). A total of 156 patients with BCLC B1 stage were enrolled in the study, of whom 25 underwent LR and 16 underwent radiofrequency ablation; the results demonstrated that the median OS time of patients with BCLC B1 stage was 4.3 years, which was similar to the results of the present study.

Taken together, radical treatment may provide better survival outcomes compared with TACE in patients with BCLC B1 subclass. However, whether LR should be recommended as a first-line treatment for patients with BCLC B stage remains controversial. Wada et al. discussed the selection criteria for LR in patients at BCLC B stage (20). They divided the patients into three groups according to the tumor burden; results showed that patients with up to three lesions (<5 cm) had a significantly improved survival rate compared with other patients treated with LR; moreover, the 3- and 5-year survival rates were 87.4% and 75.2%, respectively, which were comparable with the survival rates reported in the present study. However, the recurrence outcomes in our study were notably improved compared with those reported by Wada et al.; the 3- and 5-year PFS rates in our study were 54.3% and 45.7%, respectively, while those in Wada et al.'s study were 34.4% and 18.8%, respectively. Considering the differences in long-term outcomes, patients in the present study had a lower microscopic intrahepatic metastasis and a lower average serum AFP level. As demonstrated by numerous studies, microvascular invasion and high preoperative tumor marker levels were reported to be independent risk factors for postoperative recurrence (21, 22).

Recently, Chen et al. used the Markov model to compare the efficacies of LR and TACE for BCLC stage B1. They simulated a randomized controlled trial (RCT) with a follow-up period of 15 years. The median OS was 43.3 months, with the 3- and 5-year survival rates of 41.3% and 30.6%, respectively. Patients were recruited between 2008 and 2014. Subsequent advancements in surgical concepts and equipment have validated the benefits of LR compared with TACE for BCLC stage B. Taken together, LR is a potentially curative therapy, whereas TACE is a palliative therapy that leaves viable tumor cells in the liver tissue. Thus, whether the indication for LR in selective patients with BCLC B stage should be expanded remains controversial. Hence, high-quality RCT and systematic reviews should be performed to resolve this controversy. A recent systematic review supported the role of LR as a treatment option for BCLC B HCC, and emphasized the need to refine the criteria for LR (23).

The present study focused on a subclass of BCLC B HCC, BCLC B1, which includes patients with favorable liver function and low tumor burden. According to the definition of BCLC B1, the tumor burden was beyond the Milan criteria but within the up-to-7 criteria. Among all the patients who underwent LR, 25 (71.4%) presented with no more than two tumors and were good candidates for LR, either lobe resection or segment resection. A total of five patients had tumor lesions located in both lobes, two of whom underwent right semi-hepatectomy plus regional left liver lobe resection (one tumor lesion located in the inferior left lateral lobe). The other three patients underwent anatomic segment resection plus radiofrequency ablation. In addition, two patients presented with tumor lesions located at the long distal to the liver capsule, while one patient presented with tumor lesions proximal to the large hepatic vein; all three patients were classified as having unresectable HCC. Therefore, these patients did not undergo liver transplants. It is well known that the incidence of HCC and disease-related mortality remains high in China; approximately 50% of new-onset HCC cases worldwide are diagnosed in China every year. A large number of patients are on the waiting list to receive a liver transplant. As the indications for liver transplantation in HCC patients remain limited, the majority of medical centers in China still recommend the Milan Criteria as the golden standard for selecting HCC patients who require a liver transplant. In our clinical practice, patients diagnosed with BCLC B1 stage HCC have not yet been identified as candidates for liver transplantation. With regard to the safety of LR, no perioperative surgery-related deaths were observed among the enrolled patients; meanwhile, several patients experienced bile leakage, pulmonary infection, and liver failure, and only two patients developed severe postoperative complications (grade 3/4).

Both surgery and TACE require good liver function based on the Child–Pugh score or albumin–bilirubin grade, due to the risk of liver function deterioration after treatment. The percentage of patients with a Child–Pugh score of 7 (Child class B) was comparable between the LR and TACE groups. According to current guidelines, only patients with a Child class A liver function are candidates for LR and TACE. However, previous studies have demonstrated the safety and efficacy of LR and TACE therapy in patients with HCC and Child class B HCC (24, 25). Although Child class B classification is a negative risk factor for the survival of patients treated with LR and TACE, the negative risk factor should not be considered an absolute contraindication and should be challenged with the balance between the potential benefit and the possibility of liver function deterioration (26). In the present study, 10 patients with a Child–Pugh score of 7 underwent LR and TACE, and no postoperative mortality was reported. Among them, two patients had severe postoperative complications (grade 3–4). The available data probably revealed the safety and efficacy of LR and TACE for selected patients (Child score of 7) with Child class B. As regarding the impact of an underlying liver disease on HCC recurrence after treatment, the etiology of HCC differs between different regions worldwide. In China, approximately 90% of all HCC cases are derived from chronic HBV infection—liver cirrhosis—HCC, also known as trilogy. However, alcohol consumption and hepatitis C virus (HCV) infection are the primary causes of HCC in Europe and North America. The treatment of underlying liver disease has important impact on HCC recurrence and prognosis after radical or regional therapies (27–30). In our clinical practice, patients with HBV infection receive oral entecavir treatment daily throughout their lifetime; if tolerance occurred, tenofovir fumarate was used as antiviral therapy. Alcohol abuse has been recognized to play a potential role in the incidence and recurrence of HCC, and previous studies have revealed its prognostic impact in patients with HBV-related liver disease (31). According to the World Health Organization, alcohol abuse is defined as daily alcohol intakes of >25 mg in men and >15 mg in women. To decrease the incidence and recurrence of HCC, we recommend alcohol abstinence in all patients once diagnosed.

This study had several limitations. First, this was a nonrandomized controlled study conducted at a single center; therefore, a selection bias may exist. Second, the sample size of this study was small and not representative of all patients with HCC. Therefore, large-scale, multicenter, randomized controlled studies are required.

In conclusion, LR significantly prolonged the OS time and reduced the recurrence rate (with the risk reduced by 59%) of patients with BCLC B1 stage compared with TACE. In addition, tumor lesions distributed in both lobes of the liver and treatment strategy (LR vs. TACE) were independent risk factors for OS and PFS. Tumor lesions distributed in both lobes of the liver increase the risk of mortality (by four fold) and postoperative recurrence (by threefold).

The results of the present study favor the Kinki criteria over the Bolondi's criteria, suggesting that LR can benefit patients with BCLC B1 stage compared with TACE. Taken together, the results of the present study indicate that LR is associated with increased OS and PFS compared with TACE in patients with BCLC B1 HCC.

## Data Availability

The original contributions presented in the study are included in the article/Supplementary Material, further inquiries can be directed to the corresponding author.
